# Surgical management of glaucoma: Evolving paradigms

**DOI:** 10.4103/0301-4738.73692

**Published:** 2011-01

**Authors:** Tarek Sharaawy, Shibal Bhartiya

**Affiliations:** University of Geneva Hospitals, 22 Rue Alcide Jentzer, 1211 Genève 14, Switzerland

**Keywords:** Cyclodestructive procedures, glaucoma drainage devices, nonpenetrating glaucoma surgery, trabeculectomy

## Abstract

Surgical intervention is mandatory in the case of documentation of the progression of glaucomatous optic neuropathy despite the administration of maximal tolerated medical therapy, and in cases where compliance is poor. Minimal complications, good long-term intraocular pressure (IOP) control, and precisely titrated target IOPs resulting in avoidance of visual impairment are the primary goals of surgical intervention. This article is an attempt to provide a broad overview of the therapeutic options available to the glaucoma surgeon. The available surgical modalities have undergone modifications and refinements over time, with a view to improve patient outcomes and visual recovery, yet are fraught with intra- and postoperative complications. The risk and benefits of each of the available surgical options must be critically evaluated and customized to fit the needs of the particular patient. There is insufficient evidence at present to establish the superiority of any of these surgeries over the other.

Intraocular pressure (IOP) reduction remains the mainstay of glaucoma therapy, with evidence that medical, laser, and surgical techniques all are providing a similar mean, long-term, daytime IOP control and optic disc and visual field stability.[1–3] The IOP fluctuation during the day and the water-drinking test has been found to be significantly greater in the medically treated group compared with the surgical group.[4,5] Surgery for glaucoma is therefore indicated when optimum medical therapy and/or laser surgery fails to sufficiently lower IOP or there is sufficient evidence that the patient does not have access to or cannot comply with medical therapy. In case of angle closure glaucomas, failure of a peripheral iridectomy to relieve the pupillary block, as well as peripheral anterior synechiae involving more than 180° of the angle may be considered as indications for surgery.

A risk-benefit analysis on a case-to-case basis is essential, considering both the structural and functional integrity of the optic nerve and the progression of optic neuropathy, before a decision is made to resort to surgery.

An estimation of risk of progression, the physical quality of life and the life expectancy of the patient all affect the threshold for surgical intervention. To select a particular surgical procedure, the individual target pressure for the respective patient has to be defined, and an informed decision taken, in consultation with the patient, keeping in mind all the determinants of surgical success and complications. With evidence from randomized case control trials and the introduction of newer surgical modalities, as of course, the surgical refinements in the old, perhaps the time is right for a reevaluation of the glaucoma treatment paradigm.[6,7]

## Trabeculectomy

Trabeculectomy, though long established as the gold standard in glaucoma filtering surgery, is fraught by numerous short and long term complications including bleb leaks, infections, accelerated cataract progression, choroidal effusions, hemorrhage, and prolonged or permanent visual impairment from hypotony maculopathy. The optimal surgical algorithm for trabeculectomy is yet to be one of consensus, but the following modifications have proven to be safe and effective in most patients.[7–10]

**Figure 1:**
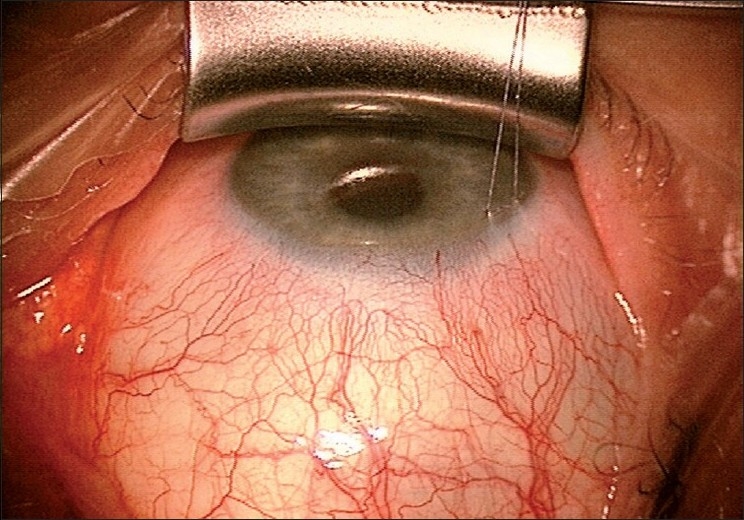
Corneal traction suture

The preferred site for the bleb is superior, nasal, or temporal, leaving one of the two sites free for a repeat procedure, if required. A corneal traction suture [Fig. 1] provides adequate exposure for a fornix-based flap with extensive blunt dissection, preventing “ring of steel” scars. A partial thickness scleral flap dissected 1 mm into clear cornea, limiting the side incisions at 0.5 mm from the cornea, results in a more diffuse, posterior bleb. Mitomycin C (MMC) in concentrations of 0.2 and 0.4 mg/ml is recommended for 1–3 minutes, customized to each case. A 0.5-mm punch for the trabeculectomy and the use of an anterior chamber maintainer (ACM), together with two adjustable and two releasable sutures for the scleral flap help in better titration of the IOP.

### Review of the literature

Stalmans *et al*. studied the long-term effects of Khaw’s technique and reported an IOP less than 18 mmHg in 90.9%, and an IOP less than 14 mmHg in 61.4% subjects.[9] Postoperative complications were infrequent: flat AC (1.8%), hypotony (1.5%), choroidal detachment (8.9%), and wound leak (0.5%).[10] The ACM enables the titration of flap sutures: at 30-cm bottle height, a flow rate of 1 drop/s would correspond to an IOP of 10-15 mmHg postoperatively.[9,10] The results of this technique have been found to compare favorably with published data using the Watson’s modification of Cairn’s technique both in terms of IOP control and complications.

The use of antifibrotics is associated with a higher incidence of complications while avoiding their use results in a much higher short-term failure rate. Results from Collaborative Initial Glaucoma Treatment Study (CIGTS) indicated that primary trabeculectomy with or without antifibrotics was equivalent to medical therapy for open angle glaucoma through 5 years of follow-up, with IOPs comparable to those after newer surgical procedures, such as the trabectome and canaloplasty. Although trabeculectomy was seen to result in lower IOPs than laser and medical therapy, visual field outcomes were judged to be equivalent.[3]

Lee *et al*. concluded that there is a complex interaction of patient and surgical variables, and inadequate evidence at the present time exists to claim superiority for any MMC protocol, with or without titration.[11]

The Tube versus Trabeculectomy (TVT) Study has challenged several current concepts about the safety and efficacy of trabeculectomy: tube-shunt surgery was reported to have a much higher success rate during the first 3 years of follow-up, comparable IOP reduction after 3 months, and no difference in medication use at 3 years. The incidence of postoperative complications was higher after trabeculectomy with MMC, but serious complications associated with vision loss and/or reoperation, including cataract, developed with similar frequency after both surgical procedures.[12,13] Regardless, trabeculectomy with minimal modifications on a case-to-case basis definitely offers the possibility of tailoring the postoperative IOP in cases of glaucoma refractory to medical therapy. A reduced rate of complications due to surgical refinements and precisely titrated target IOPs can radically modify the visual outcome of trabeculectomy.

## Nonpenetrating Glaucoma Surgery

Nonpenetrating surgical procedures improve the safety profile of glaucoma surgery, and maybe considered at an earlier stage of the disease, especially when medical or laser treatment is insufficient or unavailable.

Deep sclerectomy (DS) has emerged as the forerunner in this regard with its superior safety profile.[14] Following DS, the aqueous outflow is enhanced by removing the inner wall of Schlemms canal and juxtacanalicular trabecular meshwork (TM), with the intact trabeculo-Descemets membrane (TDM) controlling the aqueous outflow through the filtration site. The second technique, viscocanalostomy (VC), relies on the patency of putative aqueous exit channels, enhanced by dilating Schlemm’s canal using a high-density viscoelastic substance. In sinusotomy, a band of sclera is removed parallel to the limbus exposing the Schlemm’s canal. However, the inner wall of Schlemm’s canal and TM are left intact. *Ab externo* trabeculectomy consists of the removal of the inner wall of Schlemm’s canal with the juxtacanalicular TM and the covering of the sclerectomy site with a superficial scleral flap.

These procedures may be indicated in both primary and secondary open angle glaucomas, and especially in uveitic glaucoma, as they induce less inflammation, and in high myopia and Sturge–Weber syndrome, which are at a higher risk for choroidal detachment.[15–18] Absolute contraindications include neovascular glaucoma and ICE syndrome. The surgery is relatively contraindicated in eyes with narrow angles and with damaged trabeculum (e.g., posttraumatic angle recession, postlaser trabeculoplasty).[14]

### Surgical techniques

#### Deep sclerectomy

**Figure 2:**
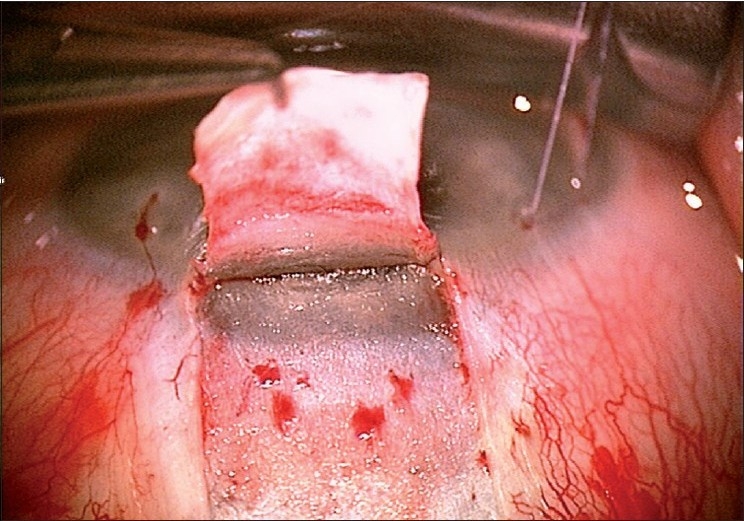
Superficial sclera flap

**Figure 3:**
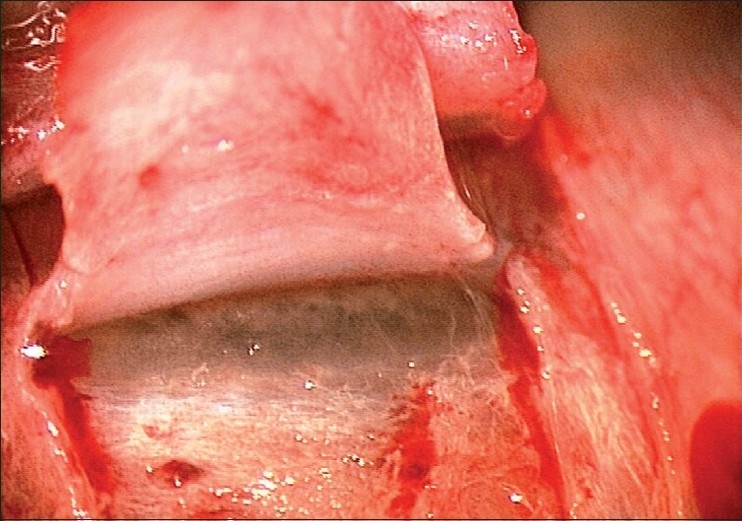
Deep sclera flap

**Figure 4:**
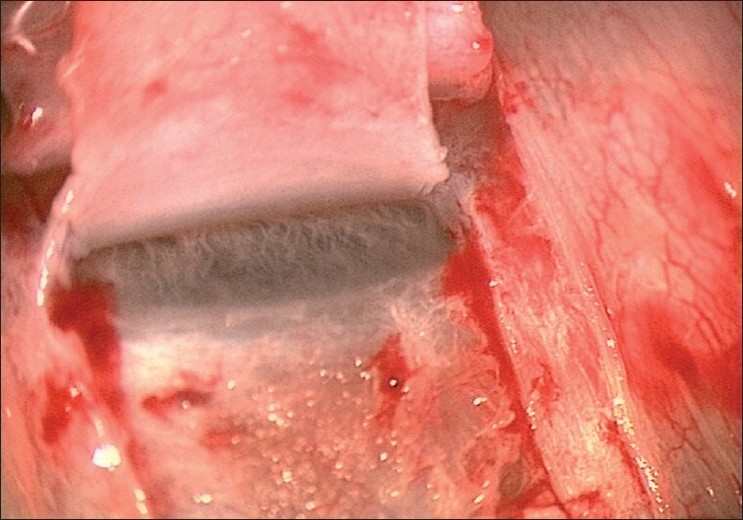
Creation of trabeculodescemetic window

**Figure 5:**
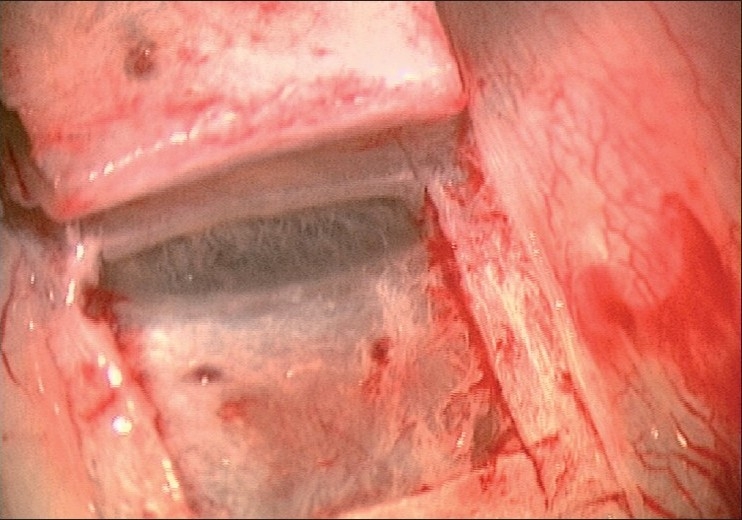
Removal of the deep sclera flap

**Figure 6:**
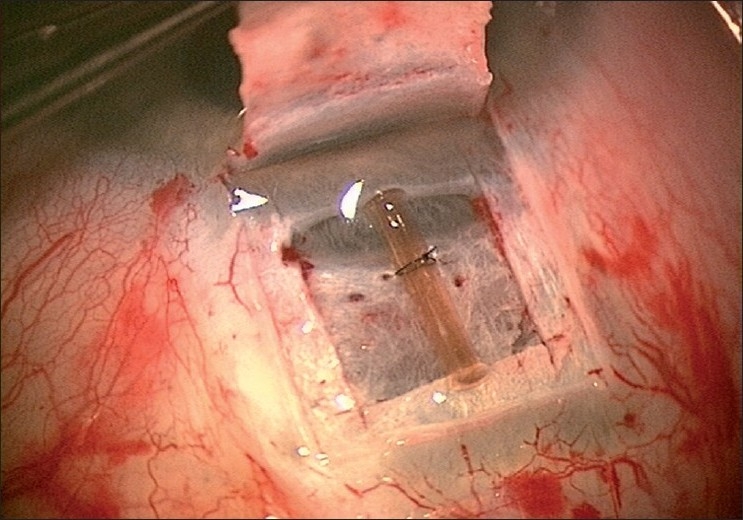
Collagen implant sutured to the sclera bed

**Figure 7:**
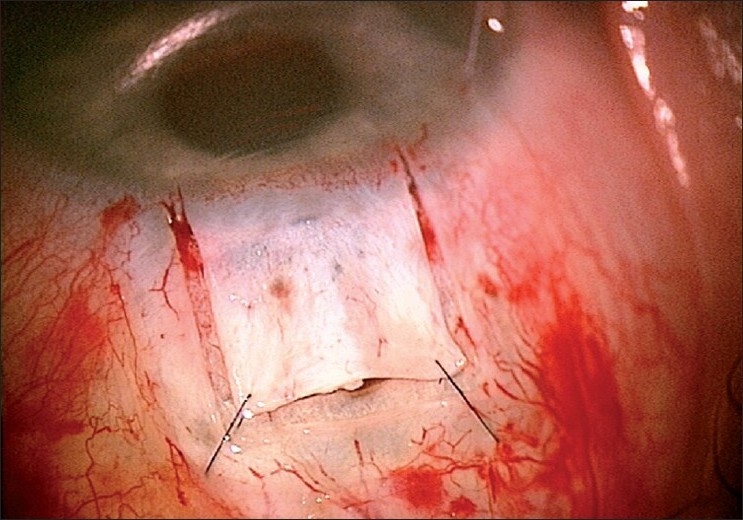
Superficial flap sutured

A limbus-based conjunctival flap is made, and an antimetabolite is applied subconjunctivally, followed by copious irrigation. A limbus based superficial 5 × 5 mm scleral flap (one-third scleral depth) is fashioned and extended 1.5 mm into the clear cornea [Fig. 2]. A second deep 4 × 4 mm scleral flap is dissected leaving only a 50- to 100-μm-thick scleral bed [Fig. 3]. The Schlemm’s canal gets deroofed as the dissection is advanced anteriorly at this critical depth. The TDM is fashioned by extending the dissection up to 1–1.5 mm into the clear cornea, taking care to peel off the Descemet’s membrane in order to prevent an inadvertent perforation [Fig. 4]. The deep flap is then excised [Fig. 5], and at this stage, the aqueous can be seen to percolate through the trabeculum. The inner wall of Schlemm’s canal and the juxtacanalicular TM are then peeled off using fine forceps. An implant may be sutured to the scleral bed to act as a spacer device during the initial healing period [Fig. 6]. The superficial scleral and conjunctival flaps are then closed with 10/0 nylon and vicryl sutures, respectively [Fig. 7].

#### Viscocanalostomy

After creation of the TDM, high-viscosity hyaluronic acid is injected into the two surgically created ostia of Schlemm’s canal, aiming at dilating both the ostia and the canal. Hyaluronic acid is also placed on the scleral bed as a spacer, and the superficial scleral flap is sutured tightly with 10-0 nylon in order keep the viscoelastic substance *in situ*, and to force the aqueous percolating through the TDM into the two ostia.

#### Review of the literature

Khairy *et al*. reported a success rate (IOP less than 22 mmHg without medication) of 61.4%, 36.6%, and 18.9% at 12, 24, and 30 months, respectively, following DS without an implant or antimetabolite.[18]

The best results of DS have been obtained by putting an implant in the scleral lake in order to keep it open. Shaarawy *et al*. compared DS in one eye and DS with collagen implant (DSCI) in the other, and found complete success at 48 months in 38.5% of DS eyes and 69.2% of eyes after the latter.[19] Bissig *et al*. reported 47.7% and 88.9% qualified and complete success, respectively, with a laser goniopuncture in 59.8% of patients after 10 years of follow-up.[20] Kozobolis *et al*. reported that 72.5% of DS achieved qualified success versus 95% of DS augmented with 0.02 mg/ml of MMC.[21] Choudhary *et al*.’s view that nonpenetrating glaucoma surgery (NPGS) augmented with small-volume MMC/5FU provided good long-term IOP control in eyes at high risk of failure with a lower incidence of complications compared with augmented trabeculectomy.[22] Others however failed to show a significant benefit of using an antimetabolite, reporting high failure rates of the procedure with and without MMC.[23]

Randomized prospective studies have found success rates of trabeculectomy and DS to be comparable.[23–26] El Sayyad *et al*. reported an IOP reduction of 15.6 ± 4.2 and 14.1 ± 6.4 mmHg, with complete success rates of 79% and 85% in DS and trabeculectomy, respectively.[24] Cillinio *et al*. found no significant difference in outcomes between DS and trabeculectomy, but were of the view that trabeculectomy could be more suitable for higher IOP levels or longer life expectancies given its higher probability of success over time.[26] DS has been found to result in less endothelial cell loss (3.3% and 5.2%), in comparison to trabeculectomy (7–14%).[27,28]

A meta-analysis of the efficacy of nonpenetrating trabecular surgery for open angle glaucoma revealed that the pooled complete success rates were DS, 69.7%; DSCI, 59.4%; DS with reticulated hyaluronic acid implant, 71.1%; and viscocanalostomy, 72.0%; with an overall weighted complete success rate of 67.8%.[15] Another meta-analysis by Hondur *et al*. found that the percentage of cases achieving IOP ≤ 21 mmHg was 48.6% after primary DS, 68.7% after DS with an implant, 67.1% after DS with an antimetabolite, 51.1% after primary VC, and 36.8% after VC with an antimetabolite or implant. With lower set IOP targets, the rates of success varied between 35% and 86% for DS, and between 10% and 67% for VC, implying that their potential to achieve lower target IOPs seems to be low.[29] A meta-analysis of 10 randomized controlled trials comparing VC and trabeculectomy by Chai *et al*. found a mean IOP difference of 2.25, 3.64, and 3.42 mmHg at the end of 6, 12, and 24 months, respectively. They concluded that trabeculectomy had a greater pressure-lowering effect compared with VC, but the latter had a significantly better risk profile.[30]

Forty-two percent of patients in the trabeculectomy group had a successful outcome compared to 21% in the VC group, out of the 50 eyes recruited for a prospective randomized control trial. The IOP was reported to be lower in the former, needing less postoperative topical IOP-lowering medication.[31]

Guedes *et al*. assessed the IOP peaks, the amplitude of variation of IOP after the water-drinking test in patients with a nonpenetrating DS and trabeculectomy, and found that the IOP variations and peaks were similar in both groups (12.3 and 14.1 ± 1.7; 10.0 and 11.6 ± 1.6 mmHg).[32]

A recent study by Mansouri *et al*. compared the quality of diurnal IOP control and IOP fluctuation during a water-drinking test in 20 patients each with trabeculectomy and DSCI. The authors reported that mean IOP for trabeculectomy (10.1 mmHg) and DSCI (13.7 mmHg) were comparable, with similar IOP fluctuations. Also, during the water-drinking test, the IOP change was 2.4 and 3.8 mmHg, respectively, for the trabeculectomy and DSCI groups, respectively.[33]

**Figure 8:**
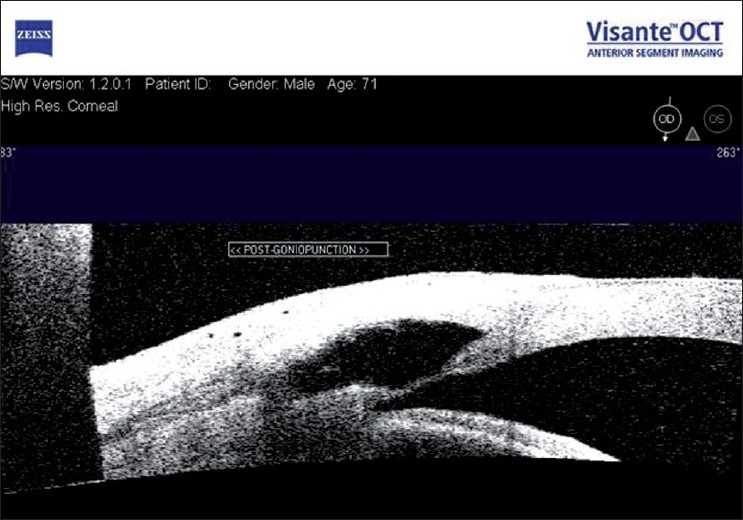
AS-OCT image after deep sclerectomy and goniopuncture

There is sufficient evidence that NPGS achieves good IOP control in the early postoperative period, and is known to have high long-term failure rates. Though its efficacy has been thought to be inferior to trabeculectomy, adjunctive techniques like intraoperative use of antimetabolites and implants, as well as laser goniopuncture [Fig. 8] have been shown to increase efficacy. Because of their superior complication profile, and further refinements in surgical techniques, NPGS is emerging as a viable surgical option especially in cases where the target IOP is not in the lower teens.

## Glaucoma Drainage Devices

Traditionally, glaucoma drainage devices (GDDs) are reserved for patients with a high risk of failure for filtering surgery, and limited to cases of refractory glaucoma. Table 1 provides a brief summary of the available GDDs.

**Table 1:** An overview of the glaucoma drainage devices currently in use

Device	Material	Shape	Comment
Valved			
Ahmed	Polypropylene	Pear	Plate area: 184 mm^2^ (FP7)
			Pars plana variant with a clip plate (PC 7) Venturi valve
Optimed	Polymethyl methacrylate	Rectangle	Plate area: 140 mm^2^
			Microtubules
Krupin disc	Sialistic	Oval	Plate area: 180 mm^2^
			Slit valve
Krupin band (360°)	Sialistic	Rectangle	Plate area: 300 mm^2^
Joseph Hitchings (360°)	Silicone	Rectangle	Plate area: 765 mm^2^
White	Silicone	Round	Plate area: 280 mm^2^
Nonvalved			
Baerveldt	Silicone	Curved	Plate area: 250, 350, 425, 500 mm^2^
Molteno	Silicone	Round	Single plate, plate area: 134 mm^2^
			Double plate, Plate area: 268 mm^2^
Schoket band (360°)	Silicone	Rectangular	Plate area: 300 mm^2^
Newer devices			
SOLX Gold shunt GMS Plus	24 karat gold	3 × 6 mm rectangle	Contains numerous micro-channels that bridge the anterior chamber and the suprachoroidal space, thus controlling the aqueous outflow
ExPress mini shunt	Stainless steel	Shaft with a spur and an external plate with a scleral slot	Size 400 μm, external diameter 27 G.
			Acts by shunting the aqueous out of the anterior chamber into the subcojunctival space
T-flux implant	poly-MEGMA, hydrophilic acrylic	T-shaped	Drains fluid by means of capillarity and osmosis
			Designed for use with nonpenetrating glaucoma surgery

The Tube versus Trabeculectomy (TVT) Study demonstrated that at 3 years, IOP was not different statistically, being 13.0 ± 4.9 and 13.3 ± 6.8 mmHg, and the number of glaucoma medications being 1.3 ± 1.3 and 1.0 ± 1.5 in the tube and trabeculectomy group, respectively. The cumulative probability of failure during the first 3 years of follow-up was 15.1% in the tube group and 30.7% in the trabeculectomy group, with postoperative complications reported in 39% and 60%; surgical complications were associated with reoperation and/or loss of two or more Snellen lines in 22% and 27%, respectively.[12,13]

A meta-analysis of aqueous shunts by Minckler *et al*. revealed no advantages to the adjunctive use of antifibrotic agents or systemic corticosteroids with currently available shunts. There was insufficient evidence to assess the relative efficacy or complication rates of specific devices beyond the implication that larger surface area explants provided more enduring and better IOP control. The principal long-term complication of anterior chamber tubes was corneal endothelial failure; the most shunt-specific delayed complication reported was the erosion of the tube through overlying conjunctiva. The rate of failure was found to be approximately 10% per year, similar as for trabeculectomy.[34]

Barton *et al*. concluded that the Ahmed glaucoma valve apparently has improved the predictability of early control, while the Baerveldt glaucoma implant has a lower rate of long-term excessive encapsulation. They acknowledged that due to better predictability, and ongoing concerns regarding the safety profile of MMC trabeculectomy, shunts are increasingly being considered as the primary surgical management. The main barrier to a wider use of shunts in less complicated glaucomas is the unknown long-term effect on the corneal endothelium, an issue that has not yet been properly addressed.[35]

GDDs seem to have benefits at least comparable, if not superior, to those of trabeculectomy in the management of complex glaucomas. However, since GDDs are usually offered at the refractory stage, they have been thought to have a limited success rate. Further long-term follow-up and comparative studies are required to establish the expanding role of GDDs in current glaucoma practice.

### Newer surgical modalities

Recently developed technologies that are substantially less invasive than trabeculectomy and do not depend on external filtering bleb formation or adjunctive antifibrotic agents promise to herald a new era in glaucoma surgery. However, they are yet to demonstrate the long-term efficacy in case series with extended follow-up, or in comparison to trabeculectomy or to each other in randomized trials.

Trabectome (NeoMedix, Tustin, CA, USA) utilizes an electric spark to ablate the meshwork and inner wall of Schlemm’s canal via gonioscopic surgery, thus removing the main resistance to the aqueous outflow from the TM and the juxtacannalicular tissues. Theoretically, it has the advantage of angle surgery in children and adults, with removing of a strip of meshwork and aspiration of tissue debris reducing the inflammatory stimuli and scarring. Reflux bleeding from Schlemm’s canal during surgery is common but transient and vision-threatening complications, including cataract, have been minimal.[35,36]

IStent provides a channel for a direct transtrabecular aqueous outflow from the AC to collector channels. It is a self-retaining, titanium (6AL4V), heparin-coated device with an inlet of 80-mm internal diameter. Schlemm’s canal is identified beneath a two-layered scleral flap and a self-illuminating microcatheter threaded through Schlemm’s canal while injecting a viscoelastic substance. A 10-0 prolene suture is fixed to the end of the microcatheter and pulled around in the reverse direction, then tensioned, knotted, and left in place within the canal tenting it inward. The most common complications reported are iStent malpositioning, not always correlated with clinical failure and reflux bleeding from Schlemm’s canal after viscoelastic substance removal.[37]

The SOLX Gold shunt is a 24-karat device with dimensions of 5.2 mm length, 3.2 mm width, and 44–68 mm thickness placed into the AC over the sclera spur via a scleral incision with the posterior end positioned in the suprachoroidal space. It has several channels through its body; besides that it can be successively opened after installation via a laser applied to windows in its AC component, offering *in vivo* postoperative adjustments in the outflow. However, increased clinical experience will clarify the real potential of this device.[38]

ExPress mini-glaucoma shunt (Optonol Ltd., Neve Ilan, Israel) is a 400-μm-wide by 3-mm-long stainless steel device which provides an immediate consistent aqueous flow through a 50-μm opening that allows for the formation of a posterior, low-diffuse bleb, thus making the procedure conjunctival dependent in the superior area of the limbus.

The other three procedures are independent of the conjunctival status, and do not cause scarring sufficient to preclude subsequent conventional surgery.[38]

## Surgery for Congenital Glaucoma

The treatment of congenital glaucoma remains a challenge as the surgical options are limited by the short window of intervention available before the disease results in devastating complications. The conventional operative procedures described for congenital glaucoma are goniotomy, goniopuncture, trabeculotomy, and trabeculectomy.

### Surgical technique

#### Trabeculotomy with/without trabeculectomy

A fornix-based conjunctival and a partial thickness sclera flap is made as for trabeculectomy, followed by the application of MMC, and a 10-0 monofilament nylon suture preplaced. A superficial radial scratch incision starting from the blue zone is made and gradually deepened until Schelmm’s canal is visualized just anterior to the circumferential fibers of the scleral spur, near the posterior aspect of the limbal grey zone, and the aqueous seen to ooze out from the cut ends. The internal arm of the Harms trabeculotome is passed into the Schlemm’s canal as far as possible, using the external arm as the guide, and rotated into the AC, tearing through the TM. A trabecular block may be removed in the case of a combined procedure, and the flaps sutured as for trabeculectomy.

#### Goniotomy

Since the prerequisite for goniotomy is a clear cornea, a drop of sterile glycerine may be applied, or its epithelium denuded to aid better visualization.

The Koeppe lens is manipulated until the optimum view of the operation field is obtained (semiopaque band of the tissue just posterior to Schwalbe’s ring) and the goniotomy knife is passed, obliquely through the limbus, across the AC until the point just engages the opposite angle in the region of the band. The knife is rotated, first to one side and then to the other, cutting through the band, treating one-quarter to one-third of the circumference of the angle. The AC is then reformed with a balanced salt solution (BSS).

#### Review of literature

Fulcher *et al*. reported that glaucoma was controlled in 92.3% and 85.7% eyes with primary and secondary infantile glaucomas, respectively, with a single trabeculectomy. A control of 100% was achieved with two trabeculectomies, with no significant complications.[39] Ikeda *et al*. reported that complete and qualified successes were achieved in 63.4% and 25.9%, respectively, of the 149 eyes following trabeculotomy alone.[40] Giampiani *et al*. reported a successful outcome in 55.26% of 114 eyes following trabeculectomy with MMC in childhood glaucomas. The life-table success rates for IOP control at 24 and 60 months were 90.2% and 50.8%, respectively, with a cumulative probability of failure of 40.8% at 12 months.[41]

Schaffer evaluated 577 consecutive goniotomies and reported a complication rate of 2%. Of 287 eyes on long-term follow-up, a success rate of 76.7% was reported, being only 30% when the signs and symptoms of glaucoma were present at birth or over the age of 24 months. In contrast, one or two goniotomies cured 94% of the cases diagnosed between the ages of 1 and 24 months.[42]

Evaluating the long-term surgical and visual outcomes in 24 eyes with primary congenital glaucoma following 360° trabeculotomy and goniotomy, Mendicino *et al*. reported that IOP was successfully controlled in 92% and 58% of eyes, respectively. They concluded that 360° trabeculotomy results in excellent pressure control and is at least as successful as multiple standard procedures.[43] Zhang *et al*. evaluated the success rates in 81 eyes and reported that the same at 1, 3, 6, and 9 years after surgery were 93.94%, 66.68%, 53.88%, and 53.88%; after trabeculotomy, the success rates were 91.30%, 86.96%, 60.73%, and 37.70%, and after combined trabeculectomy trabeculotomy (CTT), they were 92.00%, 78.00%, 62.40%, and 62.40%, respectively.[44]

Campos-Mollo *et al*. reported that the cumulative probabilities of success, after performing CTT as the initial operative procedure, were 95.5% after 12 months and 78.2% after 24 months, with this rate being maintained during 15 years of follow-up.[45]

## Cyclodestructive Procedures

Cyclodestructive procedures are important in our paradigm of glaucoma therapy, as the last resort for intractable glaucomas in eyes with failed trabeculectomy or tube shunt procedures, as an emergent temporizing measure, in eyes with minimal useful vision and elevated IOP, and in eyes which have no visual potential and need pain relief.

The therapeutic window for all types of cycloablative procedures is low, that is, aggressive treatment can lead to hypotony and phthisis while conservative treatment will have no effect on IOP. The inability to titrate a predictable and reproducible response has been its drawback. Cyclocryotherapy was the most commonly used method, but has been replaced by laser cyclophotocoagulation, which causes less pain and is associated with less inflammation, hypotony, and phthisis.[46] Certain studies have demonstrated that there was no difference in the success rate between the aqueous shunts and endoscopic cyclophotocoagulation in refractory glaucoma, in fact reporting more complications in the former.[47] However, preoperative visual acuity is the most important determinant in choosing a therapeutic option in this subgroup of recalcitrant glaucoma. All other things being equal, GDDs are more commonly used for patients with better visual acuity or potential relative to cyclodestructive procedures.

Transscleral diode cyclophotocoagulation with the G-probe is the safest and most commonly used laser procedure because it is noninvasive and does not require a clear cornea or widely dilated pupil.[48] Randomized control trials are essential to define the indications and scope of cycloablation, with more refined parameters, minimizing collateral tissue damage, and newer applications of existing technology.

## Summary

The decision to perform a glaucoma surgery, and the choice of the surgical procedure, must be taken after a comprehensive risk-benefit analysis on a case-to-case basis. Modern trabeculectomy techniques, specially releasable and adjustable sutures, minimize complications of excessive filtration. The use of antifibrotics must be judicious and a long-term follow-up of these eyes is advisable. Early recognition of complications and appropriate intervention improve success and minimize patient morbidity. NPGS has proven to be an effective therapeutic option, especially when moderate lowering of IOP is required, providing an adequate peak and 24-h IOP control, with fewer and less severe complications as compared to trabeculectomy and is an important therapeutic option. GGDs are indicated in refractory glaucomas when filtering procedures are unlikely to be successful, or have failed. Their scope in primary gluacomas is also expanding given the predicitability of their results. Newer surgical procedures are yet to be validated in randomized control trials, but hold promise in their scope and safety profile. Laser diode cyclophotocoagulation with the G-probe is the last resort for refractory glaucomas when both filtration procedures and drainage implants have a high probability for failure or have failed.
